# Comparison between Scalp EEG and Behind-the-Ear EEG for Development of a Wearable Seizure Detection System for Patients with Focal Epilepsy

**DOI:** 10.3390/s18010029

**Published:** 2017-12-23

**Authors:** Ying Gu, Evy Cleeren, Jonathan Dan, Kasper Claes, Wim Van Paesschen, Sabine Van Huffel, Borbála Hunyadi

**Affiliations:** 1 Department of Electrical Engineering (ESAT), STADIUS Center for Dynamical Systems, Signal Processing and Data Analytics, KU Leuven, Leuven 3001, Belgium; sabine.vanhuffel@esat.kuleuven.be (S.V.H.); bori.hunyadi@esat.kuleuven.be (B.H.); 2 Imec, Leuven 3001, Belgium; 3 Laboratory for Epilepsy Research, University Hospital Leuven, Leuven 3000, Belgium; evy.cleeren@uzleuven.be (E.C.); wim.vanpaesschen@uzleuven.be (W.V.P.); 4 Byteflies, Antwerp 2600, Belgium; jonathan.dan@byteflies.com; 5 UCB, Brussels 1070, Belgium; kasper.claes@ucb.com

**Keywords:** seizure detection, epilepsy, EEG, EOG, wearable sensor, SVM

## Abstract

A wearable electroencephalogram (EEG) device for continuous monitoring of patients suffering from epilepsy would provide valuable information for the management of the disease. Currently no EEG setup is small and unobtrusive enough to be used in daily life. Recording behind the ear could prove to be a solution to a wearable EEG setup. This article examines the feasibility of recording epileptic EEG from behind the ear. It is achieved by comparison with scalp EEG recordings. Traditional scalp EEG and behind-the-ear EEG were simultaneously acquired from 12 patients with temporal, parietal, or occipital lobe epilepsy. Behind-the-ear EEG consisted of cross-head channels and unilateral channels. The analysis on Electrooculography (EOG) artifacts resulting from eye blinking showed that EOG artifacts were absent on cross-head channels and had significantly small amplitudes on unilateral channels. Temporal waveform and frequency content during seizures from behind-the-ear EEG visually resembled that from scalp EEG. Further, coherence analysis confirmed that behind-the-ear EEG acquired meaningful epileptic discharges similarly to scalp EEG. Moreover, automatic seizure detection based on support vector machine (SVM) showed that comparable seizure detection performance can be achieved using these two recordings. With scalp EEG, detection had a median sensitivity of 100% and a false detection rate of 1.14 per hour, while, with behind-the-ear EEG, it had a median sensitivity of 94.5% and a false detection rate of 0.52 per hour. These findings demonstrate the feasibility of detecting seizures from EEG recordings behind the ear for patients with focal epilepsy.

## 1. Introduction

Epilepsy is a serious disorder of the central nervous system that affects 1% of the world’s population [1]. Approximately 30% of epilepsy patients are not helped effectively by medication [2]. The disorder manifests itself clinically by sudden alterations in consciousness, movement, sensation, behavior, or autonomic events [3]. The unpredictable occurrences and consequences of seizures profoundly impacts the quality of life for patients and their caregivers. Accurate detection and logging of seizures are essential for the diagnosis, management and for better understanding of epilepsy as a dynamical disorder. Long-term monitoring is beneficial to increase the possibility of capturing seizures and track the evolution of the disease, thereby offering objective information on seizures. As long-term monitoring generates a lot of data, automatic seizure detection is important for a quick and objective assessment of the disorder. Automatic seizure detection could also be part of a closed-loop system for delivering treatment after detecting seizures.

Electroencephalogram (EEG) is a non-invasive recording of brain activities. It has been widely used with applications both in clinical practice and in basic and applied neuroscience [4,5]. It provides a direct measurement of spatially-aggregated neural electrical activity with high temporal resolution, which makes it convenient to accurately detect the onset of epileptic seizures. However, current EEG systems are bulky, which limit their use to a controlled environment like a hospital or lab. Patients need to be hospitalized and possibly stay for one or two weeks in order to capture seizures, which occur unpredictably and usually without warning. This approach shows limited time and cost efficiency. In order to better monitor seizures, there is a need for developing a wearable EEG system for long recording periods in a natural environment [6,7]. Moreover, long term use of wearable devices by many epileptic patients in daily life would provide researchers an effective and rich database to better reveal the long term effects of seizures.

With advances in electronic miniaturization, wireless communication and computing power, there is now an increasing interest in the development of wearable EEG sensors that provide discrete, unobtrusive, and user-friendly long duration recording solution [8,9,10,11,12,13,14,15]. For example, the work from Debener’s group has demonstrated that reliable EEG data can be recorded behind the ear with a cEEGrid electrode array, which consists of ten electrodes printed on a c-shape flexible sheet to fit around the ear. The study showed the alpha attenuation during eyes opening and P300 in auditory odd ball testing with cEEGrid, which was comparable to scalp EEG [12]. Later the group showed that the identification of the attended speaker can be achieved by cEEGrid and it has potential to be used in the brain-computer interface (BCI) steering of hearing aids [13]. Alternatively, in-the-ear EEG recording has been proposed, tested with standard EEG paradigms and benchmarked against scalp EEG recording. Alpha attenuation, auditory steady-state response (ASSR) and steady-state visually-evoked potential (SSVEP) have been observed with both a personalized earpiece and generic earpiece [11,14]. Debener’s group, in a review paper, showed an illustrative example of a few minutes of epileptiform brain activity recorded with a cEEGrid from a seven year-old boy [15]. These studies are very promising and it can be hypothesized that epileptic activity can be recorded without using traditional scalp EEG, but further systematic studies will be required to establish this. Moreover, even if this activity can be measured, it should be proven that the quality is sufficient for automatic seizure detection.

Numerous automatic seizure detection algorithms have been described in the literature. They mainly include two stages: feature extraction and classification [16,17,18,19,20,21,22]. The epileptic EEG is characterized by its spectral, temporal, and spatial distribution. The relevant features have been extracted by various methods, including FFT, autoregressive modeling, wavelet transform, phase synchronization, entropy, spatial filtering, and convolution kernels. The features extracted from multiple channels can be integrated in several ways. The early integration concatenates features from each channel into one long vector, which is used to train a classifier [21]. The late integration trains the classifier for each channel, then combines the outcomes of channels into a final decision [22]. The nuclear norm learning approach constructs a feature-channel matrix to preserve inherent spatial characteristics of the EEG [20]. In a patient-specific seizure detector, developed by Shoeb and Guttag [19], besides concatenation of features from each channel, they also encoded time evolution by concatenating feature vectors from contiguous and non-overlapping 2 s segments to form one long feature vector.

The objective of the present study was to prove the feasibility of automatic seizure detection with unobtrusive EEG electrodes placed behind the ear. To the best of our knowledge, this is the first study on seizure detection using EEG recorded behind the ear. Traditional scalp EEG with four additional electrodes placed behind the ear were simultaneously recorded from epilepsy patients in the hospital. We first investigated the potential to record epileptic EEG behind the ear and compared its quality with traditional scalp EEG. In addition, we conducted automatic seizure detection and compared the detection performance using scalp EEG with EEG recorded behind the ear.

## 2. Materials and Methods

First, we describe the experiment. We will then elaborate upon the methods for evaluating the quality of EEG recorded behind the ear and seizure detection.

### 2.1. Patients

Twenty-four patients with refractory focal epilepsy, who were admitted for long-term video-EEG recording as part of a pre-surgical evaluation, participated in the study. Five did not have seizures during long-term VideoEEG monitoring in the hospital, seven had seizures, but without EEG correlates, and twelve had focal onset impaired awareness seizures with ictal EEG changes. These twelve patients (six females) were included in the study. Ten of them had temporal lobe epilepsy (TLE) and two had extratemporal lobe epilepsy (ETLE). Mean age was 36 years old (range: 19–64). The experimental protocol was approved by the local ethical committee. All participants gave their written informed consent for the study. Detailed patients’ information is listed in Table 1.

### 2.2. Clinical EEG Recordings

Traditional multi-channel scalp EEG, which is used routinely in clinical practice, were recorded using a Schwarzer EEG amplifier (Schwarzer epas 29, Planegg, Germany) with Ag/AgCl electrodes (Ambu Neuroline Cup, Ballerup, Denmark) in the University Hospital Leuven. Scalp electrodes were placed according to the International 10–20 System [23] with additional sphenoidal electrodes. The EEG recordings were referenced to Fpz and grounded at the forehead with a sampling frequency of 250 Hz. The 22 bipolar channels used were: Fp2-F8, F8-T4, T4-T6, T6-O2, T4-Sph2, Fp2-F4, F4-C4, C4-P4, P4-O2, Fz-Cz, Cz-Pz, Pz-O2, Pz-O1, Fp1-F7, F7-T3, T3-T5, T5-O1, T3-Sph1, Fp1-F3, F3-C3, C3-P3, P3-O1. The dataset consisted of long-term EEG recordings. Patients 1, 4, and 11 had short recording times (Table 1). Experts (EC and WVP) annotated each seizure onset and end. A total of 47 seizures were captured during 431 h of hospital monitoring.

### 2.3. Behind-the-Ear EEG Setup

**Figure 1:**
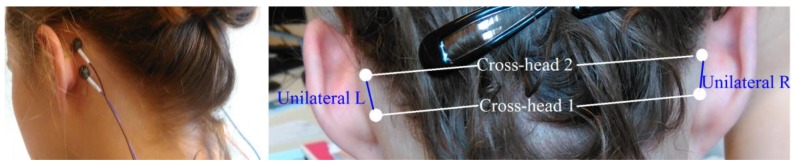
Behind-the-ear EEG setup. In the right picture, each white circle represents an EEG electrode. A line between two electrodes represents an EEG channel whose signal is derived by taking the potential difference between those two electrodes. White lines represent channels derived between the left and right ear. Blue lines represent unilateral channels.

In addition to the clinical EEG configuration, four additional electrodes (Ambu Neuroline Cup, same as above) were glued on the skin behind the ears (two each side) as shown in left photo in Figure 1, and were connected to the same clinical EEG amplifier. Using these electrodes, four behind-the-ear channels derived by taking the potential difference between left ear electrode and right ear electrode and between two electrodes behind each ear were Cross-head 1: LeftCenter-RightCenter (LC-RC), Cross-head 2: LeftTop-RightTop (LT-RT), Unilateral L: LeftTop-LeftCenter (LT-LC), and Unilateral R: RightTop-RightCenter (RT-RC), shown in the right picture in Figure 1. In what follows, we call this set of channels ‘behind-the-ear EEG’.

### 2.4. Preprocessing

Muscle artifacts were removed by applying a canonical correlation analysis for blind source separation (BSSCCA) for all channels including scalp EEG channels and behind-the-ear EEG channels [24,25]. Then the signals were band-pass filtered between 0.5 and 35 Hz. One hour long epochs around each seizure were extracted, which we call seizure epochs in the rest of the paper. For each seizure, five one-hour-long seizure-free epochs were extracted over 24 h recording, covering awake, resting state, sleep and various daily activities and are referred to as non-seizure epochs.

### 2.5. Comparison of Electrooculography (EOG) between Scalp EEG Channels and Behind-the-Ear EEG Channels

EOG resulting from eye blinking was first visually inspected and compared between behind-the-ear EEG and scalp EEG. Then EOG morphology was examined and compared based on grand average EOG.

Independent component analysis (ICA) was applied to decompose multi-channel scalp EEG signals into maximally independent components with the EEGlab ICA toolbox [26,27]. By visually inspecting the independent components, the one corresponding to EOG was identified. Then, peaks with amplitudes between 30 µV and 80 µV were detected from the time course of the independent component for EOG to detect the occurrences of EOG. Epochs (0.2 s before peaks and 0.2 s after) were extracted from the EEG signal. The epochs were averaged on the scalp channel (Fp2-F8) and all behind-the-ear EEG channels for each patient. The mean amplitude between 0.1 s before the peak and 0.1 s after was calculated from the averaged EOG for each patient and referred to as the amplitude of EOG. A Wilcoxon signed rank test was carried out to investigate whether the amplitudes of the EOG were significantly different between behind-the-ear EEG channels and scalp Fp2-F8. Outcomes were considered significant at *p*-values < 0.05.

### 2.6. Comparison between Scalp EEG and Behind-the-Ear EEG during Seizure

Power spectral density (PSD) was calculated and averaged over epochs during seizures and compared between the two recordings. Spectral coherence $C_{xy}$ was used to measure the degree of similarity between all channels from behind-the-ear EEG and all channels from scalp EEG. The coherence was averaged among epochs with ictal EEG and among epileptic discharges in the range of 2–20 Hz.

$$C_{xy}(f)=\frac{{\left|G_{xy}(f)\right|}^{2}}{G_{xx}(f)G_{yy}(f)}$$

where $G_{xy}$(*f*) is the cross-spectral density between *x* and *y*.

$G_{xx}$(*f*) and $G_{yy}$(*f*) are the auto-spectral density of *x* and *y*, respectively.

### 2.7. Seizure Detection

**Figure 2:**
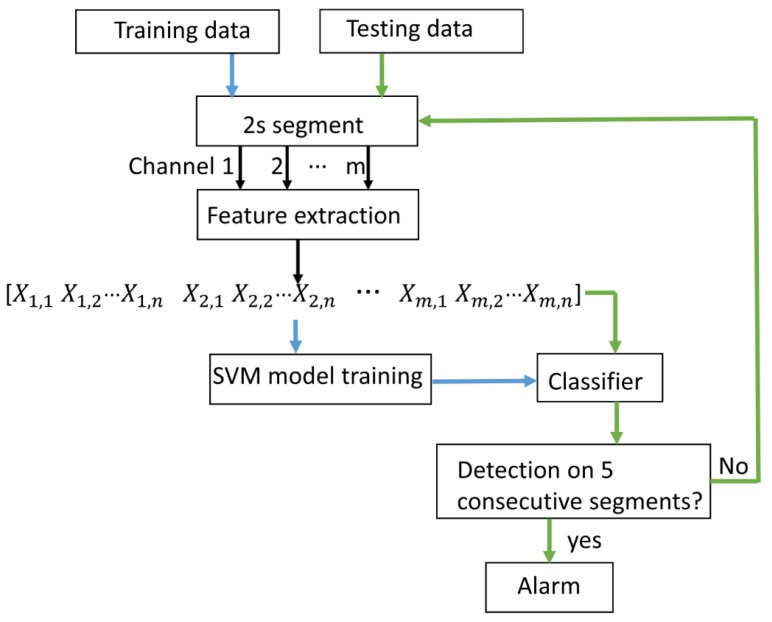
Block diagram of seizure detector training and testing (*m*: number of channels; *n*: number of features of each channel).

The seizure detection is based on features reflecting rhythmic discharges and on classification performed with a support-vector machine (SVM). Figure 2 depicts the steps of the algorithm.

#### 2.7.1. Feature Extraction

Selection of discriminative EEG features is crucial for seizure detection. In this study, 16 features per channel were extracted based on morphological characteristics of epileptic EEG. Since EEG is non-stationary, it is important to extract EEG features in a reasonably short time window in order to reflect the current underlying brain state. A two-seconds-long window was chosen as it is commonly used [19]. Fifteen mean powers covering 1–2 Hz, 1.5–2.5 Hz, 2–3 Hz, 2.5–3.5 Hz, 3–4 Hz, 3.5–4.5 Hz, 4–5 Hz, 4.5–5.5 Hz, 5–6 Hz, 5.5–6.5 Hz, 6–7 Hz, 6.5–7.5 Hz, 7–8 Hz, 8–14 Hz, and 14–20 Hz and the peak frequency were extracted from each channel. These 16 features from each channel were concatenated into one feature vector as shown in Figure 2 to capture spatial information. The size of each feature vector was 16 × 22 for scalp EEG and 16 × 4 for behind-the-ear EEG, respectively.

#### 2.7.2. SVM Classification

The SVM is a robust classification method, which has demonstrated good generalization property in various applications [19,28,29]. Since the seizure and non-seizure data are often not linearly separable [19], a non-linear SVM classifier with a radial basis function (RBF) kernel was used in this study. The central idea is to classify data from two classes by building a hyperplane from a training set. Given a training set $\left(x_{i},y_{i}\right)$, *i* = 1, …, *N* where $x_{i}\in R^{n}$ and $y_{i}=\{\pm 1\}$, $x_{i}$ is a data point and $y_{i}$ indicates the class which the point $x_{i}$ belongs to. The standard SVM requires the solution of the following optimization problem [30]:

$$\underset{w,b,\xi }{\min}\frac{1}{2}w^{T}w+c\sum_{i=1}^{N}\xi_{i}\;\mathrm{subject}\;\mathrm{to}\;y_{i}(w^{T}\varphi (x_{i})+b)\geq 1-\xi_{i},\;\xi_{i}\geq 0.$$

where the function φ maps $x_{i}$ into a higher dimensional space. *w* is the weight vector and b is the bias of the hyperplane. A slack variable ($\xi_{i}$) and a penalty parameter (*c*) are introduced if the training data cannot be separated without error. As a consequence, training samples can be at a small distance $\xi_{i}$ on the wrong side of the hyperplane. In practice, there is a trade-off between a low training error and a large margin. This trade-off is controlled by the penalty parameter *c*. The following steps were carried out for classification with the SVM:

*Kernel selection*. The Gaussian kernel $K(x,y)=\exp(-\frac{{\left\|x-y\right\|}^{2}}{2\sigma^{2}})=\varphi {\left(x\right)}^{T}\varphi (y)$ was chosen. This kernel depends only on the parameter σ.

*Cross-validation.* Leave-one-out cross-validation was applied to estimate the detection performance for each patient. The training set consisted of feature vectors computed from all non-seizure epochs and all seizure epochs except one. The seizure feature vectors were extracted from the first six seconds following a seizure onset. The testing set contained the withheld seizure epoch. The training set was further divided into subsets for optimizing penalty parameter *c* and Gaussian kernel parameter σ (inner cross-validation). The set of parameters *c* and σ were searched among positive values, with a log-scaled in the range [10^−3^, 10^3^]. The set of parameters *c* and σ corresponding to the lowest probability of error estimated from the inner cross-validation was applied to build classifier for detecting seizure in the testing set. This process was repeated until all seizures were tested. For each test, we reported whether the test seizure was detected and the number of false detections.

The performance of detection was measured in terms of sensitivity and false detection rate. Sensitivity was calculated as the number of detected seizures divided by the total number of seizures. False detection rate was calculated as the number of times a seizure was declared without an actual seizure during the one hour seizure epoch. A Wilcoxon signed rank test was carried out to investigate whether the performances in terms of sensitivity and false detection rate were significantly different between behind-the-ear EEG and scalp EEG. Outcomes were considered significant at *p*-values < 0.05.

### 2.8. Comparison of Seizure Detection between Cross-Head Channels and Unilateral Channels

Seizure detection was performed on each channel of behind-the-ear channels. Unilateral channels were assigned to either the ipsilateral side or the contralateral side relative to the epilepsy focus. A Wilcoxon signed rank test was carried out to investigate whether the performances in terms of sensitivity and false detection rate were significantly different between channels of behind-the-ear EEG. Outcomes were considered significant at *p*-values < 0.05.

## 3. Results

### 3.1. Artifacts

**Figure 3:**
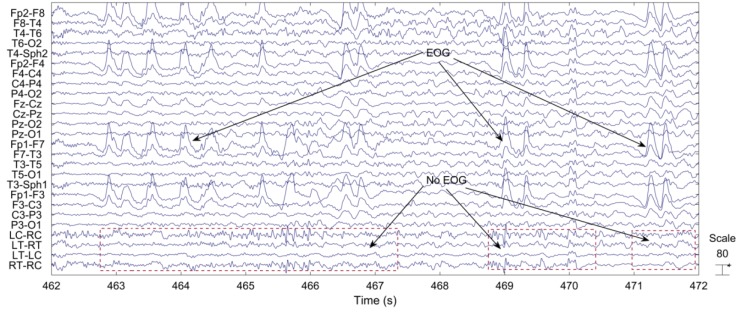
EEG segment with EOG artifacts.

Based on extensive visual inspection, we found that EOG artifacts were not visible at recordings behind the ear. See, for example, Figure 3, where the last four channels are behind-the-ear EEG channels.

**Figure 4:**
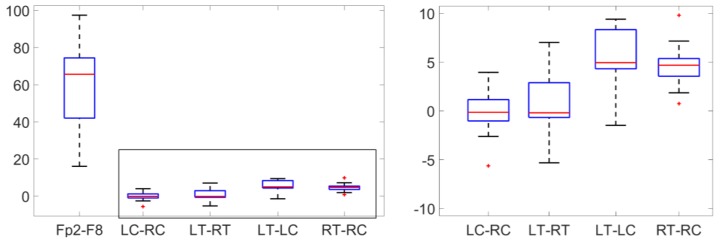
Boxplots on the left represent the distribution of amplitudes of EOG from Fp2-F8, LC-RC, LT-RT, LT-LC, and RT-RC among the patients. The right plot is a zoomed-in version of the portion indicated inside the gray rectangle in the left plot.

**Figure 5:**
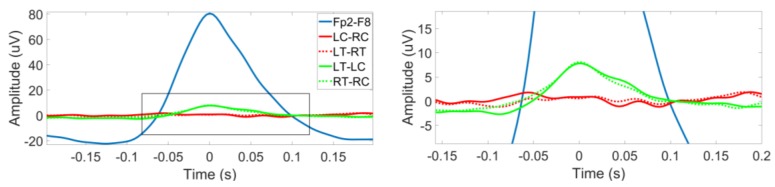
Grand average EOGs among the patients in the left. The right plot is a zoomed-in version of the portion indicated inside the gray rectangle in the left plot.

Figure 4 showed the distribution of EOG amplitudes from Fp2-F8, LC-RC, LT-RT, LT-LC, and RT-RC among the patients. The averaged amplitudes of EOG among the patients were 59.66 for Fp2-F8, −0.23 for LC-RC, 0.62 for LT-RT, 5.50 for LT-LC, and 4.66 for RT-RC. Significance tests showed that EOG from Fp2-F8 had significantly higher amplitude than that from behind-the-ear-EEG channels (*p* < 0.001). Grand average EOGs from Fp2-F8 and behind-the-ear EEG channels among the patients were showed in the Figure 5. The zoomed-in plot showed that the EOG from LT-LC and RT-RC had similar morphology to that from Fp2-F8 and had significantly smaller amplitudes than that from Fp2-F8. The EOG was absent from LC-RC and LT-RT.

### 3.2. Comparison of Scalp EEG and Behind-the-Ear EEG during Seizure

**Figure 6:**
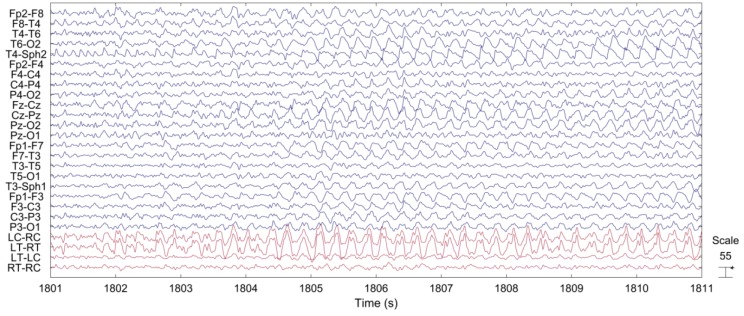
Time series of representative scalp EEG and behind-the-ear EEG during seizure.

Figure 6 shows a representative example of the ictal EEG of patient 3. The sustained rhythmic activity can be clearly observed on both scalp EEG channels and on behind-the-ear EEG channels.

**Figure 7:**
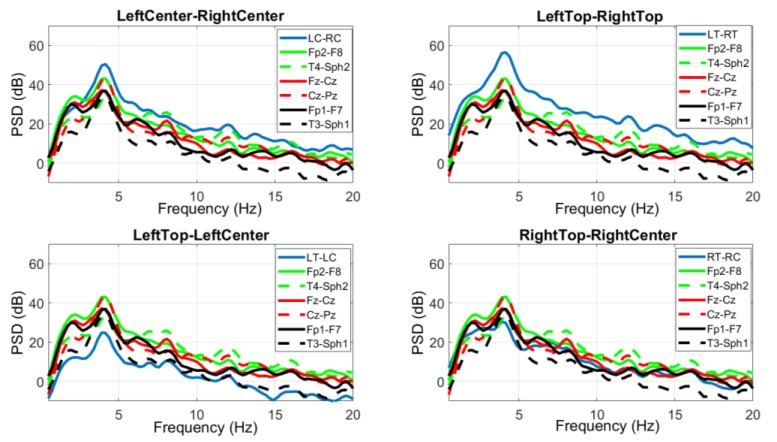
Averaged PSD of scalp EEG and behind-the-ear EEG during seizures.

Figure 7 shows the PSD from selected scalp EEG channels and each of the behind-the-ear channels. The PSD was calculated during a 10s seizure period starting from seizure onset. The frequency content and pattern is similar between these two recordings. The peak at ~4 Hz is related to the ictal pattern.

Spectral coherence has been calculated between each behind-the-ear EEG channel and each scalp EEG channel and was averaged among seizures. The best matchup scalp channel with respect to the behind-the-ear channel was the one with highest coherence value. Table 2 above shows the best matchup scalp channel to each ear EEG channel and the corresponding coherence value on 12 patients. It can been seen that most best matchup scalp channels were the scalp channels which were nearby behind-the-ear EEG channels as seen in Table 2. The averaged coherence among patients for LC-RC, LT-RT, LT-LC and RT-RC with their best matchup scalp EEG channel were 0.83, 0.83, 0.82, and 0.80, respectively. The coherence (≥0.80) indicated that behind-the-ear EEG channels record meaningful epileptic activities similarly to scalp EEG.

### 3.3. Seizure Detection

**Figure 8:**
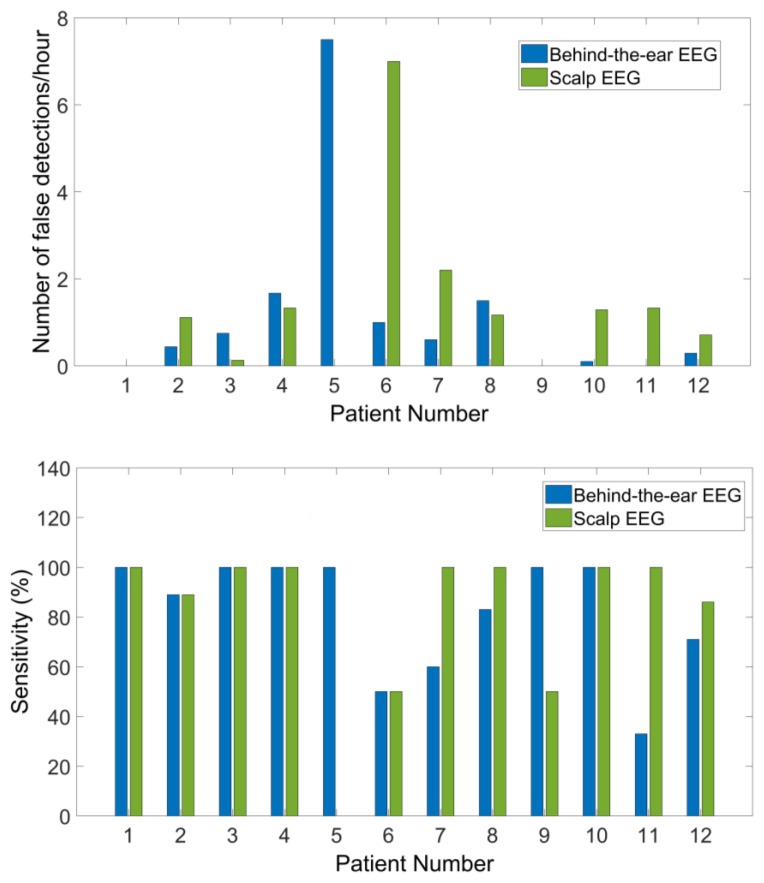
False detection rates and sensitivities of seizure detection among the patients.

**Figure 9:**
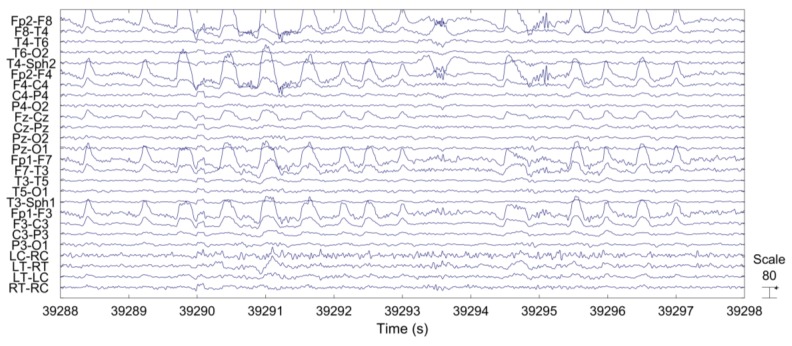
Example of repetitive EOG artifacts causing false detections from scalp EEG and no false detections from behind-the-ear EEG on patient 10.

**Figure 10:**
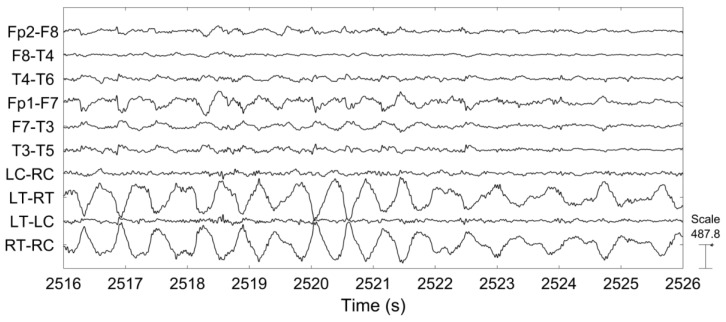
Example of abnormal EEG causing false detections from patient 2.

**Figure 11:**
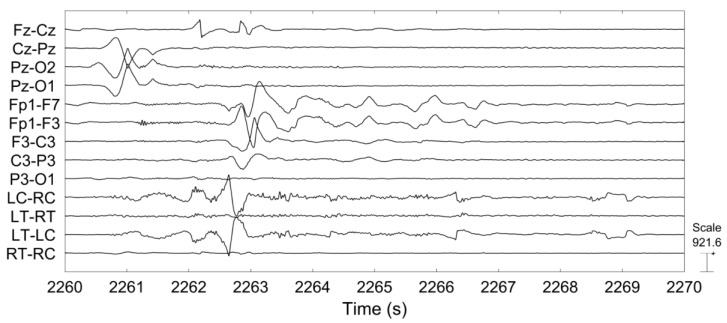
Example of abnormal EEG causing false detections from patient 4.

Figure 8 shows the number of false detections declared per hour and the sensitivity for each patient from both recordings. Table 3 reports the central tendency of detection performances among 12 patients by median and mean. Detection performances varied significantly among those patients and had a skewed distribution. Therefore, we use the median, which provides a more robust data descriptor for skewed distributions to report our results. Among 47 seizures, 41 seizures were detected from scalp EEG and 38 from behind-the-ear EEG. The median sensitivity for scalp EEG is 100% with a false detection rate of 1.14 per hour. Median sensitivity for behind-the-ear EEG is 94.50% with a false detection rate of 0.52 per hour. Detectors performed well on most patients from both recordings. Patient 1 and patient 9 had no false detections. Patient 5 had 100% detection from behind-the-ear EEG, but no detection from scalp EEG. For this patient, after seizure onset, the eye blinking patterns (EOG artifacts) were quite different between seizures. As presented above, EOG artifacts resulting from eye blinking were absent at LC-RC and LT-RT and had very small amplitudes at LT-LC and RT-RC where EOG can only be observed after average of EOG epochs. Therefore, the disturbance of EOG was negligible on behind-the-ear EEG. This might contribute to the fact that detection from behind-the-ear EEG was better than that from scalp EEG. For patient 6 and patient 10, the detection sensitivities were the same on both recordings. However, there were more false detections from scalp EEG than from behind-the-ear EEG. Those false detections were caused by repetitive EOG artifacts which contaminated some scalp EEG channels. Figure 9 shows an example of repetitive EOG artifacts causing false detections from scalp EEG, with no false detections from behind-the-ear EEG on patient 10. Inconsistent spatial distribution between seizures from patient 9 might be the reason for the 50% seizure detection sensitivity of scalp EEG. Figure 10 and Figure 11 showed examples of the artifacts which caused false detections for patient 2 and patient 4, respectively. Both were electrode artifacts. In Figure 10, scalp channel Fp1-F7 and behind-the-ear EEG channels LT-RT and RT-RC had rhythmic activities with abnormally high amplitudes. This was due to poor electrode contact during eating. The phase reversal between LT-RT and RT-RC indicated the RT electrode had poor contact. In Figure 11, the artifacts were caused by poor electrode contact. A Wilcoxon signed rank test showed that there was no significant difference on performance between those two recordings in terms of sensitivity (*p* = 1) and false detection rate (*p* = 0.32).

### 3.4. Comparison of Seizure Detection between Cross-Head Channels and Unilateral Channels

**Figure 12:**
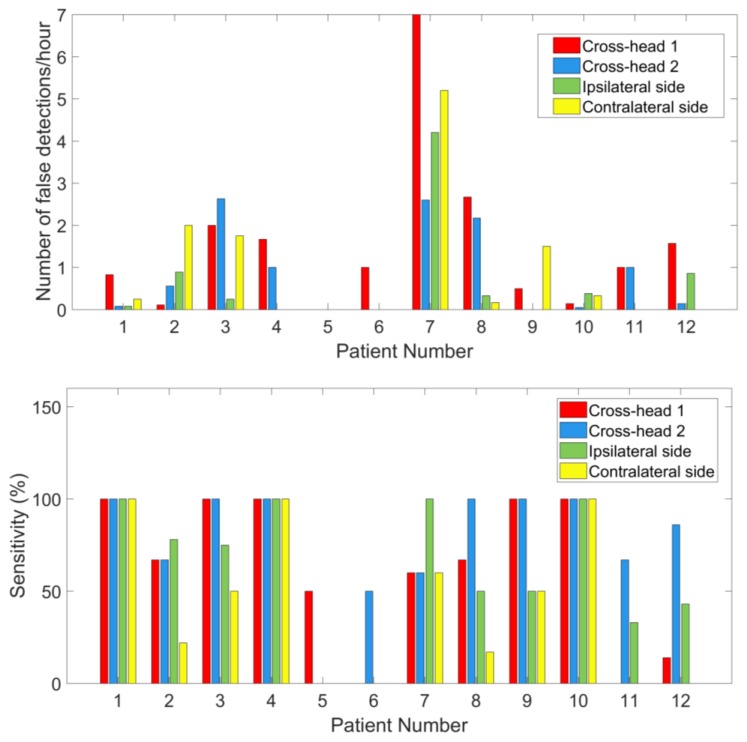
False detection rates and sensitivities of seizure detection from cross-head channels and unilateral channels among the patients.

**Figure 13:**
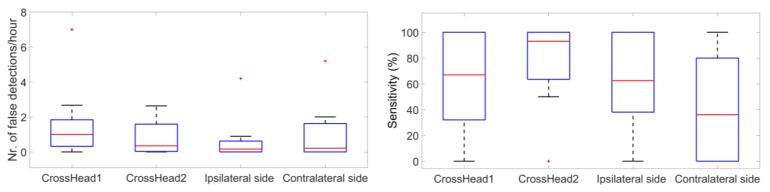
Boxplots representing distribution of false detection rates and sensitivities from cross-head channels and unilateral channels among the patients.

Figure 12 shows false detection rates and sensitivities of seizure detection from cross-head channels and unilateral channels among the patients. The performance varied greatly among patients. For patients 1, 4, and 10, cross-head channels and unilateral channels showed the same sensitivity of 100%. Patients 2 and 7 showed the best sensitivity on the ipsilateral side. Figure 13 shows the distribution of false detection rates and sensitivities of behind-the-ear channels among the patients. Statistical tests have shown that contralateral side had statistically lower sensitivity than cross-head 1 (*p* = 0.03), cross-head 2 (*p* = 0.02), and the ipsilateral side (*p* = 0.03). The false detection rates were statistically different between cross-head 1 and cross-head 2 (*p* = 0.04) and between cross-head 1 and the ipsilateral side (*p* = 0.01).

## 4. Discussion

In order to continuously monitor epilepsy patients and detect their seizures in daily life, the recording setup should be unobtrusive and hidden from view to other people. In this study, we evaluated the possibility to record epileptic EEG and detect seizures from a few electrodes placed behind the ear, which present a new and innovative approach for seizure detection. The aim is to utilize wearable sensors which record EEG behind the ear for our wearable seizure detection system in the future.

The behind-the-ear EEG was simultaneously recorded with scalp EEG, using the same ground and reference electrodes. The quality of behind-the-ear EEG was validated by comparison with scalp EEG. The analysis of EOG artifacts from eye blinking has shown that EOG artifacts had negligible amplitudes on unilateral channels RT-RC and LT-LC and were absent on cross-head channels LC-RC and LT-RT. Note that the absence of EOG artifacts from eye blinking on the cross-head channels is due to the fact that the electrode pair for cross-head behind-the-ear EEG channels were placed symmetrically with respect to the eyes. Therefore EOG artifacts resulting from eye blinking were not recorded. It is expected that EOG artifacts from eye blinking are also absent on cross-head scalp channels in which the electrode pair are symmetrically placed with respect to the eyes, for example T5–T6. A previous study showed suppression of EOG artifacts with in-the ear EEG recording [9]. It is advantageous that EOG artifacts from the recordings behind the ear were absent or had very small amplitudes, which can only be observed from averaged many EOG artifact epochs. EOG artifacts are the cause for poor performance of automatic detection. Repetitive eye blinks may be mistaken as a seizure by an algorithm, causing false detections. This was the case in patient 6 and patient 10, where more false detections were caused by EOG artifacts from scalp EEG. EOG artifacts will also obscure the seizure activity causing missed seizures. Patient 5 had no seizures detected from scalp EEG due to different eye blinking patterns after seizure onset between seizures. With recording behind the ear, there may not be the need for development of EOG artifact removal or correction methods. This will reduce the computational complexity and reduce power requirements, which are very useful for a real-time seizure detection system in a portable device with limited size.

The epileptic EEG was examined and compared between behind-the-ear EEG and scalp EEG in the time and frequency domains. The time waveform of behind-the-ear EEG resembled that of scalp EEG as shown in Figure 6. A comparison in the frequency domain, through comparison of the PSD, also shows a strong match between behind the ear and scalp EEG (see Figure 7). Further, coherence analysis was performed to investigate the similarity between behind-the-ear EEG and scalp EEG during a seizure. Coherence values (≥0.80) between each behind-the-ear channel and the best matchup scalp EEG channel indicated that behind-the-ear EEG recorded similar epileptic discharges to its counterpart scalp EEG. Cross-head behind-the-ear EEG channels had a higher amplitude of epileptic activities than the unilateral behind-the-ear EEG channels shown in Figure 6 and Figure 7. This is due to the fact that focal seizures are often asymmetric [3]. Using cross-head recordings, it involves wires from both sides. This can mean a design challenge for a truly unobtrusive device. Results from a comparison of seizure detection between cross-head channels and unilateral channels have shown that seizure detection performance of those channels varied greatly among patients. Patients 1, 4, and 10 had the same sensitivity of 100% among those four channels. Patients 3, 9, 11, and 12 had better sensitivities from cross-head channels than the ipsilateral side. The significance test showed that cross-head channels had comparable performance of seizure detection with the ipsilateral side. Cross-head channels and the ipsilateral side had statistically significantly higher sensitivities than the contralateral side. The finding showed that cross-head channels can be selected for patients with focal epilepsy for seizure detection. With prior knowledge of the epileptic foci side, the unilateral channel from the epileptic foci side can be selected in order to develop a more compact, wearable device.

Seizure detection has been carried out separately for behind-the-ear EEG and scalp EEG. Our method reached a median sensitivity of 100% for scalp EEG with a false detection rate of 1.14 per hour, with a median sensitivity of 94.50% for behind-the-ear EEG with a false detection rate of 0.52 per hour. A variety of seizure detection methods have been reported in the literature [20,22,31,32]. By taking the seizure morphology into account, Meier’s algorithm detected 96% of seizures with a false detection rate of 0.45 per hour [31]. Shoeb and Guttag reported detection performance with a sensitivity of 96% and a false detection rate of 0.08 per hour by applying a machine learning technique [19]. By using a self-organizing neural network, Gabor detected 92.8% seizures with 1.35 false alarms per hour [33]. In our method, the features covering delta (1–4 Hz), theta (4–8 Hz), alpha (8–14 Hz), and beta (14–20 Hz) and peak frequency were arranged into a long feature vector. Most of the time, the frequency of epileptic EEG resides in delta and theta bands. Therefore, in order to pick up frequency information as precisely as possible in those two bands, we extracted averaged power within a 1 Hz window overlapped with 0.5 Hz. We chose averaged powers during 8–14 Hz and 14–20 Hz to represent information in the alpha and beta bands, respectively. At this moment, all those features were used for classification. In the future, the results might be improved by feature selection methods to reduce redundancy and improve relevance [34]. Other features will be also considered, for example, the features reflecting the synchronization of neurons firing [35].

There is no significant difference in performance of seizure detection between those two recordings. When there was not much EOG artifact contamination, scalp EEG showed better performance because of its rich spatial information, as shown in patients 7, 8, 11, and 12. With many eye blinking artifacts, behind-the-ear EEG had better performance, as shown in patient 5, patient 6, and patient 10. Some false detections were caused by poor electrode contact. Poor contact results in instability of impedance, leading to sharp or slow waves with varying morphology, as shown in Figure 11. If a poor contact is under the situation of rhythmic movement, such as eating, the artifacts look rhythmic, as shown in Figure 10. It was observed that electrode artifacts resulting from poor contact happened sparsely in time and occurred only in one or several channels. Late integration for classification, which is to carry out separate detection on individual channels and then combine the outcomes of channels using a voting scheme, will be attempted in order to find a way of reducing the influences from channels with electrode artifacts. Additionally, sparse time artifact removal [36] will be examined to remove electrode artifacts.

Cortically-generated potentials, like epileptic EEG, have a physiological distribution characterized by a maximum potential at the source and then gradually dropping off in voltage with increasing distance across the scalp. This is due to the volume conduction effect [37]. Behind-the-ear EEG channels are close to some channels located in the temporal lobe; therefore, epileptic EEG with TLE was well captured by behind-the-ear EEG channels in this study. For ETLE, epileptic EEG originates from remote lobes, and behind-the-ear EEG is expected to pick it up due to volume conduction or the spreading of the ictal activity. In this study, only two patients with ETLE were included, one patient with parietal lobe epilepsy and one patient with occipital lobe epilepsy. No patients with frontal lobe epilepsy (FLE) were recorded during the study. Therefore we cannot provide evidence that epileptic EEG with FLE will be recorded by behind-the-ear EEG channels.

Detecting seizures from behind-the-ear recording in this paper is original. To the best of our knowledge, this is the first study to report successful seizure detection using electrodes placed behind the ear. If incorporated in a wearable device, such a behind-the-ear EEG setup may provide an unobtrusive method for monitoring epilepsy patients in daily life, which is socially acceptable and a key requirement for a wearable system. The contribution of the paper is a proof that seizure detection can be achieved with four behind-the-ear channels comparably with scalp EEG. Key features, such as four-channel recording and negligible disturbance by EOG artifacts from eye blinking, will reduce algorithm complexity and, consequently, consume less battery power, which is very important for wearable systems. Behind-the-ear EEG can be extended to other applications, where long-term recording in daily life is required and limited spatial resolution is sufficient, for example BCI based on P300, SSVEP, or alpha oscillation and sleep staging.

## 5. Conclusions

The study provides substantial evidence that behind-the-ear EEG can capture high-quality epileptic activities for patients with TLE and, to a lesser extent, patients with ETLE from the parietal lobe and occipital lobe. Seizures can be successfully detected for those patients with behind-the-ear EEG. The performance of detection was comparable to scalp EEG. The main advantage with a few behind-the-ear recordings is that it offers potential for an unobtrusive, ambulatory, and easy to use system. This study presents an important step forward in the development of a wearable seizure detection system.

## Figures and Tables

**Table 1:** Patients’ information (PID: patient ID; AED: anti-epileptic drug).

PID	Nr. of Seizures	Sex	Age	Seizure Onset Zone and AED Dosage on the Inspection Day	Recording Time (h)
1	1	F	19	Right occipital lobe Topiramate (100 mg)	12
2	9	F	24	Left temporal lobe Levetiracetam (2000 mg) Clobazam (10 mg)	91
3	8	M	32	Right temporal lobe Carbamazepine (500 mg)	52
4	1	M	64	Left temporal lobe Lamotrigine (200 mg) Carbamazepine(200 mg) Lacosamide (200 mg)	3
5	2	M	61	Right temporal lobe Lamotrigine (200 mg) Levetiracetam (2000 mg)	27
6	2	F	33	Right parietal lobe No AED	23
7	5	M	45	Left temporal lobe Lamotrigine (200 mg) Perampanel (2 mg)	34
8	6	F	32	Left temporal lobe Lamotrigine (200 mg) Levetiracetam (2000 mg)	72
9	2	F	49	Left temporal lobe Lacosamide (100 mg)	43
10	1	M	28	Right temporal lobe Topiramate (100 mg) Lamotrigine (200 mg)	21
11	3	F	25	Right temporal lobe Lamotrigine (225 mg) Levetiracetam (1250 mg)	10
12	7	M	20	Left temporal lobe Lacosamide (350 mg) Perampanel (4 mg) Lamotrigine (400 mg) Oxcarbazepine (300 mg)	43

**Table 2:** Coherence between behind-the-ear EEG channel and the best matchup scalp EEG channel on 12 patients (SD: Standard Deviation; PID: Patient ID).

PID	LC-RC		LT-RT		LT-LC		RT-RC	
1	Fz-Cz	0.96	F4-C4	0.99	F7-T3	0.99	T4-Sph2	0.89
2	T5-O1	0.91	T5-O1	0.92	T5-O1	0.93	T3-T5	0.79
3	Fp2-F8	0.81	Fp2-F8	0.82	T3-Sph1	0.75	C4-P4	0.81
4	F7-T3	0.95	F4-C4	0.96	F3-C3	0.93	F3-C3	0.74
5	T4-Sph2	0.88	T4-T6	0.85	Cz-Pz	0.74	T4-T6	0.81
6	Cz-Pz	0.81	T3-Sph1	0.73	C3-P3	0.79	Fp2-F8	0.78
7	T5-O1	0.71	T4-Sph2	0.68	C3-P3	0.73	P4-O2	0.87
8	T3-T5	0.70	T3-T5	0.73	F3-C3	0.76	T6-O2	0.79
9	P3-O1	0.91	F7-T3	0.95	T3-Sph1	0.95	T4-Sph2	0.92
10	Fp2-F4	0.94	Fp2-F4	0.87	Fp1-F3	0.85	P4-O2	0.81
11	Fp1-F7	0.77	T5-O1	0.77	P4-O2	0.71	Pz-O1	0.70
12	T5-O1	0.65	T6-O2	0.68	F3-C3	0.65	T4-Sph2	0.66
Mean ± SD	0.83 ± 0.11		0.83 ± 0.11		0.82 ± 0.11		0.80 ± 0.07	

**Table 3:** Averaged detection performance among the patients (min: minimum value; max: maximum value; SD: standard deviation; Ear EEG: behind-the-ear EEG).

	False Detections/h		Sensitivity (%)	
	Scalp EEG	Ear EEG	Scalp EEG	Ear EEG
Median (min max)	1.14 (0 7)	0.52 (0 7.50)	100 (0 100)	94.50 (33 100)
Mean ± SD	1.36 ± 1.91	1.15 ± 2.08	81.25 ± 31.76	82.17 ± 23.40
